# Impact of Medical Community Model on Intravenous Alteplase Door-to-Needle Times and Prognosis of Patients With Acute Ischemic Stroke

**DOI:** 10.3389/fsurg.2022.888015

**Published:** 2022-04-27

**Authors:** Hongfei Li, Dongjuan Xu, Yunyun Xu, Lianyan Wei

**Affiliations:** Department of Neurology, Dongyang People's Hospital, Affiliated to Wenzhou Medical University, Dongyang, China

**Keywords:** medical community model, acute ischemic stroke, tissue-type plasminogen activator, intravenous alteplase, door-to-needle time

## Abstract

**Objective:**

In this study, we retrospectively analyzed 795 AIS patients who received intravenous alteplase for thrombolytic therapy in one third-class hospital or three second-class hospitals in Dongyang City and sought to evaluate the effects of the medical community model on intravenous alteplase door-to-needle time (DNT) and prognosis of patients with acute ischemic stroke.

**Methods:**

According to whether the medical community model is established or not, 303 AIS patients (204 cases from the third-class hospital and 99 cases from three second-class hospitals) were assigned to control group unavailable to the medical community model and 492 AIS patients (297 cases from the third-class hospital, and 195 cases from three second-class hospitals) into observational group available to the medical community model.

**Results:**

A higher thrombolysis rate, a shorter DNT, more patients with DNT ≤ 60 min and DNT ≤ 45 min, a shorter ONT, lower National Institutes of Health Stroke Scale (NIHSS) scores at 24 h, 7 d, 14 d, and modified Rankin scale (mRS) scores at 3 months after thrombolytic therapy, a shorter length of hospital stay, and less hospitalization expense were found in the observational group than the control group. Subgroup analysis based on different-class hospitals revealed that the medical community model could reduce the DNT and ONT to increase the thrombolysis rate of AIS patients, especially in low-class hospitals. After the establishment of the medical community model, the AIS patients whether from the third-class hospital or three second-class hospitals exhibited lower NIHSS scores at 24 h, 7 d, 14 d after thrombolytic therapy (*p* < 0.05). After a 90-day follow-up for mRS scores, a significant difference was only noted in the mRS scores of AIS patients from the third-class hospital after establishing the medical community model (*p* < 0.05). It was also found that the medical community model led to reduced length of hospital stay and hospitalization expenses for AIS patients, especially for the second-class hospitals.

**Conclusion:**

The data suggest that the medical community model could significantly reduce intravenous alteplase DNT and improve the prognosis of patients with AIS.

## Introduction

Stroke is a leading cause of death worldwide and a huge risk factor for disability. As reported in 2004, 15 million people suffered from stroke annually, including 5 million deaths and another 5 million permanent physical disabilities (1). Despite strengthened prevention and better control of risk factors such as smoking and blood pressure control. From 1990 to 2016, the overall incidence rate of stroke decreased, but more than 13 million people were newly diagnosed with stroke every year (2). The incidence of stroke increases with age and people aged 65 and over are at higher risk of stroke. In general, younger age groups under 50 cause a greater burden of stroke, especially in low and middle-income countries (3). Stroke is classified into five subtypes including ischemic stroke, hemorrhagic stroke, subarachnoid hemorrhage, cerebral venous thrombosis, and spinal cord stroke. Ischemic stroke is caused by a blockage of blood vessels that reduces the blood supply to a certain area of the brain (4).

Ischemic stroke is the most common subtype of stroke worldwide (5). The occurrence of ischemic stroke is associated with unalterable factors such as age, gender, and ethnicity, and accumulating risk factors such as unhealthy diet, alcohol consumption, smoking, hypertension, and physical inactivity increase stroke incidence (6). The intervention of intravenous thrombolysis (IVT) with tissue-type plasminogen activator (tPA) has been well established in patients with acute ischemic stroke (AIS). Intravenous alteplase as a recombinant tissue plasminogen activator (rtPA) is commonly used in the treatment of AIS (7, 8). The earlier IVT treatment stroke patients get, the greater the possibility of a good prognosis. Door-to-needle time (DNT) refers to the time from arrival at the hospital emergency room to the beginning of IVT. It is used to evaluate the quality of AIS care (9) and prognosis (10). Therefore, reducing the median DNT is the basic goal in the treatment of AIS patients. Hospital factors such as delayed diagnosis and the inability to determine eligibility are the leading reasons for extended DNT (11, 12).

Reduction in time for intravenous thrombolysis treatment increases the possibility of good results. Actually, previous studies have indicated that the establishment of new models, such as the Royal Melbourne Hospital thrombolysis model (13) and the Helsinki stroke thrombolysis model (12) for stroke patients might address hospital factors affecting increased DNT. In China, the majority of AIS patients had their first visits to second-class hospitals or community hospitals. Lack of experienced neurologists, equipment, and technologies due to hospital-level variation limited the rapid delivery of thrombolytic therapy rapidly to AIS patients after hospital admission, which may increase the risk of neurological deficits and poor prognosis (14). Accordingly, a new therapy model, known as the medical community model, has been developed to avoid considerably varied hospital-level outcomes for AIS patients in China. This study attempted to analyze the effect of the medical community model on DNT and the prognosis of AIS patients.

## Methods

### Study Participants

At the end of December 2018, the medical community model covering one third-class hospital (Dongyang People's Hospital Affiliated to Wenzhou Medical University) or three second-class hospitals in Dongyang City of Zhejiang Province (China) has been established and implemented. There were 4,287 AIS patients who were admitted into either one third-class hospital (*n* = 2,552) or three second-class hospitals (*n* = 1,735) in Dongyang City between January 2017 and December 2018, and all of them were unavailable to the medical community model. There were 5,306 AIS patients who were admitted into either one third-class hospital (*n* =2,961) or three second-class hospitals (*n* =2,345) in Dongyang City between January 2019 and December 2020, and all of them were available to the medical community model. All in all, from January 2017 to December 2020, a total of 795 AIS patients had received intravenous alteplase for thrombolytic therapy in one third-class hospital (Dongyang People's Hospital Affiliated to Wenzhou Medical University) or three second-class hospitals in Dongyang City. According to whether the medical community model is established or not during patient admission, 303 AIS patients admitted the hospital between January 2017 and December 2018 were assigned into a control group; 492 AIS patients admitted to the hospital between January 2019 and December 2020 were assigned into an observational group. There were 204 cases from the third-class hospital and 99 cases from three second-class hospitals among 303 AIS patients unavailable to the medical community model and there were 297 cases from the third-class hospital and 195 cases from three second-class hospitals among 492 AIS patients available to the medical community model. The diagnosis of AIS was made according to the guidelines for diagnosis and treatment of AIS initiated by the Chinese Stroke Association (CSA) (15). AIS patients undergoing thrombolytic therapy eligible for recruitment must (1) be aged 18 years or older; (2) have the admission be the first for stroke during the study period; (3) be treated with intravenous rt-PA within 4.5 h of symptom onset; and (4) have a documented DNT. AIS patients undergoing thrombolytic therapy were excluded if they (1) experienced severe head trauma within the previous 3 months; (2) received arterial puncture at a noncompressible site within the previous 7 days; (3) had previous or current intracranial hemorrhage, such as cerebral parenchymal hemorrhage, intraventricular hemorrhage, subarachnoid hemorrhage, subdural/epidural hematoma; (4) had elevated blood pressure (systolic blood pressure ≥ 180 mmHg or diastolic blood pressure ≥ 100 mmHg); (5) had active bleeding or platelet counts <100 × 10^9^/L; (6) underwent oral anticoagulants therapy, with INR > 1.7 or PT > 15 s; (7) were diagnosed with intracranial tumor or massive intracranial aneurysm; (8) underwent intracranial or intraspinal surgery within the previous 3 months; (9) underwent low-molecular weight heparin therapy within the previous 24 h; (10) had oral administration of thrombin inhibitor or factor Xa inhibitor within 48 h; (11) had a level of blood glucose <2.8 mmol/L or 22.22 mmol/L; (12) showed CT or MRI signs of > 1/3 middle cerebral artery infarctions; (13) were treated with a concomitant therapy with intra-arterial reperfusion techniques; or (14) were transferred to another acute care hospital, left against medical advice, or without a documented site of discharge disposition.

### Before the Establishment of the Medical Community Model

Those who received intravenous alteplase for thrombolytic therapy in either one third-class hospital or three second-class hospitals in Dongyang City between January 2017 and December 2018 when the medical community model was absent were classified into the control group. At that time, AIS patients experienced a series of prehospital first aid processes before receiving thrombolytic therapy and some of them failed to receive rapid delivery of thrombolytic therapy resulting from the first visit to the hospital lack of thrombolytic therapy leading to delay in hospitalization.

### After the Establishment of the Medical Community Model

Those who received intravenous alteplase for thrombolytic therapy in either one third-class hospital or three second-class hospitals in Dongyang City between January 2019 and December 2020 when the medical community model has been in service were classified into the observational group. The medical community model requires (1) an expert team with 10 neurologists including; (2) regular group learning knowledge about the guidelines for diagnosis and treatment of AIS initiated by the CSA and relevant scales used to evaluate AIS, training operation procedures for thrombolytic therapy, and communication for consensus on plan for thrombolytic therapy among team members; (3) optimization of thrombolytic therapy process, including modular training protocols for neurologists of medical community model, regular assignment of chief physicians to low-class hospitals belonging to medical community model for outpatient service and guidance, and designation of associate chief physicians and specialists into low-class hospitals belonging to medical community model as director or important members; (4) monthly regular training delivered by specialists of the medical community model, focusing on AIS, intravenous thrombolysis, rapid transfer, risk factors influencing DNT, how to reduce DNT and increase thrombolysis rate; and (5) constant optimization of thrombolytic therapy process under the medical community model, including rapidly handling 120 emergency phones, availability of neurologists for patients with suspected stroke within 15 min after arrival, availability of results of routine blood tests, blood biochemistry, coagulation analysis, and ECG examination within 45 min after arrival, CT examinations for initial diagnosis concurrent with patient data collection from family members within 25 min after arrival, initiation of intravenous thrombolysis in the absence of intracranial hemorrhage once the informed consent was obtained, and a series of examinations including National Institutes of Health Stroke Scale (NIHSS) scores, blood pressure, heart rate, and blood glucose.

### Protocols for Intravenous Alteplase

All patients fulfilled indicators for IV-tPA administration (16) and were treated with intravenous alteplase (0.9 mg/kg body weight, maximum 90 mg) (Boehringer Ingelheim GmbH), with 10% of the dose given as a bolus followed by a 60-min infusion.

### Data Collection

Demographic and clinical characteristics of AIS patients including gender, age, NIHSS scores on admission, smoking status, hypertension, diabetes mellitus, previous atrial fibrillation, hyperlipidemia, coronary heart disease, previous stroke (not within the previous 3 months), family history of cardiovascular diseases, hyperhomocysteinemia, systolic pressure, and diastolic pressure, thrombolysis rate, DNT, the proportion of patients with DNT ≤ 60 min and DNT ≤ 45 min, onset-to-needle time (ONT), National Institutes of Health Stroke Scale (NIHSS) scores 24 h, 7 days and 14 days after thrombolytic therapy with the purpose to evaluate the severity of neurological deficits, death or discharge, modified Rankin scale (mRS) scores at 3 months after thrombolytic therapy to evaluate the functional outcomes, length of hospital stay, and hospitalization expense were recorded.

The NIHSS contains 15 items of measures to evaluate the impact of AIS on different areas referring to the level of consciousness, neglect, motor strength, facial palsy, ataxia, dysarthria, and sensory loss, with scores ranging from 0 [no deficit] to 45 [most severe]. The mRS is a clinician-reported, 6-level outcome measure of global disability or dependence in the daily activities of stroke patients or other causes of neurological disability. The mRS scores range from 0 to 6, indicating a lack of symptoms to death. The mRs scores ≤ 2 at 90-day follow-up reflect good functional outcomes.

### Statistical Analysis

Data analysis was performed using SPSS 20.0 software (IBM, USA). The normal distribution of measurement data was examined by the Shapiro Wilk test. If the data fail to be normally distributed, they are expressed as median (interquartile, range) and analyzed by the Mann-Whitney *U* test otherwise the data were expressed as mean ± standard deviation and analyzed by *t*-test. The enumeration data were presented as percentages and compared using the chi-square test. If the possibility (*p*) of difference were <0.05, the difference was considered statistically significant.

## Results

### The Medical Community Model Increased the Thrombolysis Rate of AIS Patients

Among 4,287 AIS patients unavailable to the medical community model, there were 303 AIS patients who received intravenous alteplase for thrombolytic therapy in either one third-class hospital or three second-class hospitals, with the thrombolysis rate of 7.07%. Among 5,306 AIS patients available to the medical community model, there were 492 AIS patients who received intravenous alteplase for thrombolytic therapy in either one third-class hospital or three second-class hospitals, with the thrombolysis rate of 9.27%. A higher thrombolysis rate was found in the observational group than in the control group (*p* < 0.001). The control group consisted of 204 cases from the third-class hospital and 99 cases from three second-class hospitals and the observational group contained 297 cases from the third-class hospital and 195 cases from three second-class hospitals. As shown in Table 1, there was no significant difference with regard to gender, age, smoking status, hypertension, diabetes mellitus, previous atrial fibrillation, hyperlipidemia, coronary heart disease, previous stroke (not within the previous 3 months), hyperhomocysteinemia, systolic pressure, and diastolic pressure between the observational group and the control group (*p* > 0.05). Only the NIHSS scores on admission exhibited significant differences between the observational group and the control group (*p* < 0.001), showing that AIS patients had a reduced risk of neurological deficits after establishing the medical community model.

**Table 1:** Demographic and clinical characteristics of AIS patients receiving intravenous alteplase before and after medical community model.

**Characteristic**	**Before** **(*n* = 303)**	**After** **(*n* = 492)**	***p***
Gender (male/%)	169 (55.78%)	303 (61.59%)	0.105
Age (year)	69.71 ± 12.77	69.90 ± 12.90	0.840
NIHSS scores on admission	5 (3, 11)	4 (2, 9)	<0.001
Smoking status (yes/%)	53 (17.49%)	76 (15.45%)	0.448
Hypertension (yes/%)	208 (68.65%)	355 (72.15%)	0.291
Diabetes mellitus (yes/%)	54 (17.82%)	83 (16.87%)	0.730
Previous atrial fibrillation (yes/%)	50 (16.50%)	70 (14.23%)	0.384
Hyperlipidemia (yes/%)	19 (6.27%)	33 (6.71%)	0.809
Coronary heart disease (yes/%)	48 (15.84%)	70 (14.23%)	0.534
Previous stroke (yes/%)	35 (11.55%)	69 (14.02%)	0.315
Hyperhomocysteinemia (yes/%)	23 (7.59%)	23 (4.67%)	0.087
Systolic pressure (mmHg)	151.38 ± 19.75	153.86 ± 19.59	0.084
Diastolic pressure (mmHg)	84.75 ± 13.60	85.58 ± 12.92	0.389

### The Medical Community Model Reduced the DNT of AIS Patients Receiving Intravenous Alteplase

We next compared the DNT, the proportion of patients with DNT ≤ 60 min and DNT ≤ 45 min, and ONT of AIS patients between the observational group and the control group. It was found that the observational group exhibited shorter DNT and ONT, higher proportions of patients with DNT ≤ 60 min and DNT ≤ 45 min than the control group (*p* < 0.001, Table 2).

**Table 2:** The DNT and ONT of AIS patients receiving intravenous alteplase before and after medical community model.

**Item**	**Before** **(*n* = 303)**	**After** **(*n* = 492)**	***t*/χ^2^**	***p***
DNT (min)	58.16 ± 23.40	44.00 ± 18.82	9.374	<0.001
DNT ≤ 60 min (n/%)	187 (61.72%)	426 (86.59%)	8.106	<0.001
DNT ≤ 45 min (n/%)	77 (25.41%)	319 (64.84%)	10.800	<0.001
ONT (min)	145.50 ± 70.44	125.25 ± 58.12	2.089	<0.001

### The Medical Community Model Improved the Prognosis of AIS Patients Receiving Intravenous Alteplase

To investigate the effects of medical community model establishment on the prognosis of patients with AIS, we compared the NIHSS scores 24 h, 7 days, and 14 days after thrombolytic therapy, death or discharge, mRS scores at 3 months after thrombolytic therapy, length of hospital stay, and hospitalization expense of AIS patients between the observational group and the control group. As listed in Table 3, AIS patients in the observational group showed lower NIHSS scores at 24 h, 7 d, 14 d, and mRS scores at 3 months after thrombolytic therapy, a shorter length of hospital stay, and less hospitalization expense than those in the control group (*p* < 0.01). These data revealed that the establishment of a medical community model could lead to a favorable prognosis for AIS patients receiving intravenous alteplase and reduce their economic burden.

**Table 3:** The effects of medical community model establishment on the prognosis of AIS patients receiving intravenous alteplase.

**Item**	**Before** **(*n* = 303)**	**After** **(*n* = 492)**	***Z*/*t***	***p***
NIHSS scores at 24 h	4 (2, 8)	2 (1, 5)	−3.931	<0.001
NIHSS scores at 7 d	2 (0, 6)	1 (0, 4)	−3.204	0.001
NIHSS scores at 14 d	2 (0, 5)	1 (0, 4)	−2.651	0.008
mRS scores at 3 months	1 (1, 3)	1 (0, 3)	−2.592	0.010
Length of hospital stay (d)	12.43 ± 8.17	10.33 ± 5.31	4.392	<0.001
Hospitalization expense (yuan)	23,419.92 ± 20,130.52	18,532.58 ± 16,089.44	3.773	<0.001

### The Medical Community Model Increased the Thrombolysis Rate of AIS Patients Whether in Third-Class or Second-Class Hospitals

Before the establishment of the medical community model, the thrombolysis rate of the third-class hospital was 8.00% (204/2,552), which was higher than that of three second-class hospitals 5.71% (99/1,735) (*p* = 0.004). Afore the establishment of the medical community model, the thrombolysis rate of the third-class hospital was 10.03% (297/2,961), which was higher than that of three second-class hospitals 8.32% (195/2,345) (*p* = 0.036). It was also noted that the establishment of the medical community model could increase the thrombolysis rate of the third-class hospital and the second-class hospital, respectively (8.00% vs. 10.03%, *p* = 0.010; 5.71% vs. 8.32%, *p* = 0.001). No significant difference was noted concerning gender, age, smoking status, hypertension, diabetes mellitus, previous atrial fibrillation, hyperlipidemia, coronary heart disease, previous stroke (not within the previous 3 months), hyperhomocysteinemia, systolic pressure, and diastolic pressure between AIS patients from the third-class hospital and three second-class hospitals before and after the establishment of medical community model (Table 4, *p* > 0.05).

**Table 4:** Demographic and clinical characteristics of AIS patients receiving intravenous alteplase from the third-class hospital and three second-class hospitals before and after medical community model.

**Characteristic**	**Before (n** **=** **303)**		**After (n** **=** **492)**	
	**Third-class** **(*n* = 204)**	**Two-class** **(*n* = 99)**	**Third-class** **(*n* = 297)**	**Two-class** **(*n* = 195)**
Gender (male/%)	116 (56.86%)	54 (53.54%)	189 (63.64%)	114 (58.46%)
Age (year)	69.10 ± 13.05	70.97 ± 12.15	69.06 ± 13.59	71.18 ± 11.70
NIHSS scores on admission	5 (3, 10)	7 (3, 12)	4 (2, 9)	4 (2, 9)
Smoking status (yes/%)	38 (18.63%)	15 (15.15%)	48 (16.16%)	28 (14.36%)
Hypertension (yes/%)	140 (68.63%)	68 (68.69%)	207 (69.70%)	148 (75.90%)
Diabetes mellitus (yes/%)	35 (17.16%)	19 (19.19%)	44 (14.81%)	39 (20.00%)
Previous atrial fibrillation (yes/%)	39 (19.12%)	11 (11.11%)	43 (14.48%)	27 (13.85%)
Hyperlipidemia (yes/%)	9 (4.41%)	10 (10.10%)	21 (7.07%)	12 (6.15%)
Coronary heart disease (yes/%)	32 (15.69%)	16 (16.16%)	36 (12.12%)	34 (17.44%)
Previous stroke (yes/%)	23 (11.27%)	12 (12.12%)	36 (12.12%)	33 (16.92%)
Hyperhomocysteinemia (yes/%)	19 (9.31%)	4 (4.04%)	11 (3.70%)	12 (6.15%)
Systolic pressure (mmHg)	150.21 ± 19.06	153.80 ± 21.00	152.81 ± 18.91	155.46 ± 20.53
Diastolic pressure (mmHg)	83.97 ± 13.50	86.36 ± 13.73	84.99 ± 13.16	86.49 ± 12.53

### The Medical Community Model Reduced the DNT of AIS Patients Receiving Intravenous Alteplase in Third-Class or Second-Class Hospitals

We next performed a subgroup analysis to investigate the effects of the medical community model establishment on the DNT and prognosis of AIS patients receiving intravenous alteplase from the third-class hospital and three second-class hospitals (Table 5). Whether before or after the establishment of the medical community model, the three second-class hospitals showed shorter DNT and ONT, with a higher proportion of AIS patients with DNT ≤ 45 min than the third-class hospital (*p* < 0.01). These data might be explained by the phenomenon that some AIS patients regarded the third-class hospital as the first choice for admission instead of the second-class hospital, closer to their homes, leading to longer DNT and ONT in the third-class hospital than that in three second-class hospitals. After the establishment of the medical community model, the AIS patients whether from the third-class hospital or three second-class hospitals exhibited shorter DNT and ONT, with higher proportions of DNT ≤ 45 min and 60 min (*p* < 0.01), suggesting that the medical community model could reduce the DNT and ONT to increase the thrombolysis rate of AIS patients, especially for low-class hospitals.

**Table 5:** The effects of medical community model establishment on the DNT and prognosis of AIS patients receiving intravenous alteplase from the third-class hospital and three second-class hospitals.

**Item**	**Before** **(*****n*** **=** **303)**		***p***	**After** **(*****n*** **=** **492)**		***p***
	**Third-class** **(*n* = 204)**	**Two-class** **(*n* = 99)**		**Third-class** **(*n* = 297)**	**Two-class** **(*n* = 195)**	
DNT (min)	61.93 ± 19.40	50.37 ± 28.60	<0.001	46.21 ± 16.35^*^	40.73 ± 21.63^*^	0.002
DNT ≤ 60 min (*n*/%)	119 (58.33%)	68 (68.69%)	0.101	261 (87.88%)^*^	165 (84.62%)^*^	0.344
DNT ≤ 45 min (*n*/%)	29 (14.22%)	48 (48.48%)	<0.001	177 (59.60%)^*^	142 (72.82%)^*^	0.003
ONT (min)	154.75 ± 76.42	126.42 ± 51.46	<0.001	138.90 ± 68.75^*^	112.68 ± 43.42^*^	<0.001
NIHSS scores at 24 h	4 (1, 8)	4 (2, 6)	0.824	3 (1, 6)^*^	2 (1, 4.5)^*^	0.022
NIHSS scores at 7 d	2 (0, 6)	2 (0, 4)	0.253	1 (0, 4)^*^	1 (0, 3)^*^	0.059
NIHSS scores at 14 d	2 (0, 6)	2 (0, 4)	0.113	1 (0, 4)^*^	1 (0, 3)^*^	0.059
mRS scores at 3 months	1 (1, 3)	1 (0, 3)	0.307	1 (0, 3)^*^	1 (0, 3)	0.410
Length of hospital stay (d)	11.31 ± 7.40	14.75 ± 9.17	<0.001	9.89 ± 5.40^*^	11.00 ± 5.11^*^	0.023
Hospitalization expense (yuan)	24,883.84 ± 23,241.18	20,403.36 ± 10,782.09	0.069	20,629.52 ± 19,614.99	15,254.49 ± 7,113.62^*^	<0.001

* *Indicates the presence of a significant difference (p < 0.05) when before vs. after*.

### The Medical Community Model Improved the Prognosis of AIS Patients Receiving Intravenous Alteplase in Third-Class or Second-Class Hospitals

With regard to the effects of medical community model establishment on the prognosis of AIS patients receiving intravenous alteplase from the third-class hospital and three second-class hospitals, we found that only the NIHSS scores at 24 h after thrombolytic therapy exhibited a significant difference between the third-class hospital and three second-class hospital and the patients from three second-class hospitals had lower NIHSS scores at 24 h after thrombolytic therapy than those from the third-class hospital after the establishment of the medical community model *(p* = 0.022). These data might be explained by more critically ill patients with higher NIHSS scores at baseline transferred into the third-class hospital by establishing the medical community model. However, it was revealed that, after the establishment of the medical community model, the AIS patients whether from the third-class hospital or three second-class hospitals exhibited lower NIHSS scores at 24 h, 7 d, 14 d after thrombolytic therapy (*p* < 0.05), implying that the medical community model could significantly improve the prognosis of AIS patients receiving intravenous alteplase for both third- and second-class hospitals. After 90-day follow-up for mRS scores, a significant difference was only noted in the mRS scores of AIS patients from the third-class hospital after establishing the medical community model (*p* < 0.05), which may be also caused by mild-disease AIS patients arranged into the second-class hospital. Whether the medical community model was established or not, the AIS patients from the third-class hospital displayed a shorter length of hospital stay than those from three second-class hospitals, which may result from the fact that some patients from the third-class hospital would be transferred to the second-class hospitals upon disease stabilization. It was also found that the medical community model led to reduced length of hospital stay and hospitalization expenses of the AIS patients, especially for the second-class hospitals. The detailed data are listed in Table 5.

## Discussion

The treatment of AIS includes a multidisciplinary approach, which urgently needs the participation of intensive care experts. In early clinical trials, cerebral hemorrhage complications are easy to occur in thrombolytic therapy of AIS (17, 18). The first breakthrough innovation that significantly changed acute stroke care was the approval of intravenous tissue plasminogen activator (IV-tPA) by the Federal Drug Administration (FDA) in 1995 (19). IV-tPA has been the main treatment for stroke for about 25 years. For some patients with AIS, IV-tPA has proved to be an effective treatment as long as 4.5 h after onset (20). In recent years, increasing evidence indicated that every minute delay in IV-tPA is associated with worse clinical outcomes (20, 21). In this study, we focused on investigating the role of hospital factors affecting delays in IV-tPA treatment for AIS patients.

Actually, better outcomes have been achieved in some studies due to changes in medical intervention for stroke patients. Towfighi et al. (22) pointed out that intervention of team-based community health workers and advanced practice clinicians improves risk factors by controlling blood pressure after stroke, and this model was superior to usual care. A study by Sanossian et al. (23) revealed emergency medical services giving priority to sending patients with acute stroke to approved stroke centers is associated with good outcomes. It has been proven that the use of IV-tPA significantly increased (from 3.8 to 10.1%) and DNT decreased remarkably after patients with suspected stroke were sent directly to a stroke center (24). This present study implemented the usual care model (the patients received intravenous treatment in third-class or second-class hospitals in Dongyang city) and the medical community model for AIS patients in IV-tPA treatment and found that the thrombolysis rate was 7.07 and 9.27%, respectively. DNT is the main standard to evaluate thrombolytic quality of ASI. Our study discovered the patients in the medical community model showed shorter DNT and ONT than those treated with the usual care model, and there are more patients with DNT ≤ 60 min and DNT ≤ 45 min. These findings were similar to another research by Tran et al. (25), which demonstrated IVT rate of AIS patients with and without stroke team intervention was 20 and 14.4%, and the median DNT in stroke team intervention decreased 23 min. In addition, the proportion of patients with DNT ≤ 60 min and DNT ≤ 45 min was higher in stroke team intervention than that in non-intervention. Ajmi et al. (26) also manifested the median DNT decreased significantly from 27 min to 13 min after the implementation of team intervention. Although the correlation between DNT and the prognosis of ASI patients has been well-established in enormous studies, the factors related to the prognosis are different. Darehed et al. revealed each minute delay in DNT caused a series of problems including reduced survival, increased rate of cerebral hemorrhage and mobility, and worse activities of daily living (27). Since the conversion of the ischemic penumbra to irreversible infarction is time-dependent, the efficacy of IV-tPA treatment for AIS patients mainly depends on time. Early administration of IV-tPA increases the chance of saving brain injury and improves clinical outcomes (28, 29). In this study, the NIHSS scores at 24 h, 7 d, 14 d and mRS scores at 3 months after administration were lower in the AIS patients using the medical community model than those treated with the usual care model. Furthermore, the medical community model decreased length of hospital stay and hospitalization expense. Another research indicated the AIS patients receiving IV-tPA within 60 min of stroke symptom onset have a higher complete recanalization rate, showed improvement in the nervous system, and obtained better mRS scores and lower mortality at 90-day follow-up compared to the patients receiving administration after 60 min of onset (21). Tsivgoulis et al. (30) found that administration of IV-tPA within “golden hour” (60 min of symptom onset) achieved favorable functional outcome (mRS ≤ 1 at 3 months) and better clinical recovery at 2 and 24 h (reduction in NIHSS score by ≥10 points or an absolute NIHSS score of ≤ 3 at 2 and 24 h, respectively). All these findings above suggested early IV-tPA treatment and reduction in DNT were beneficial to good clinical outcomes. The subgroup analysis in the present study showed that the medical community model reduces DNT, increased the thrombolysis rate, and improves the prognosis of the AIS patients whether from the third-class hospital or three second-class hospitals.

In summary, the medical community model was helpful to the early treatment of AIS patients, which might provide the chance to reduce time in DNT and increase the thrombolysis rate, and improve clinical outcomes. However, cross-sectional multicenter studies with more than 1-year or 3-year long-term follow-up analysis should be performed to strengthen the reliability of our results. Additionally, the case data were collected from the third-class or second-class hospitals in Dongyang city and the applicability in other hospitals needs to be discussed due to hospital-level variations in medical equipment and experiences of medical staff.

## Data Availability Statement

The original contributions presented in the study are included in the article/supplementary material, further inquiries can be directed to the corresponding author/s.

## Ethics Statement

The studies involving human participants were reviewed and approved by Dongyang People's Hospital, Wenzhou Medical University. The patients/participants provided their written informed consent to participate in this study.

## Author Contributions

HFL contributed significantly to the initiation and design of the study, manuscript drafting, and manuscript editing. DJX made efforts to conception, design of the study, and manuscript revision. YYX and LYW carried out clinical data collection, data analysis, and table making. All authors approved the manuscript.

## Conflict of Interest

The authors declare that the research was conducted in the absence of any commercial or financial relationships that could be construed as a potential conflict of interest.

## Publisher's Note

All claims expressed in this article are solely those of the authors and do not necessarily represent those of their affiliated organizations, or those of the publisher, the editors and the reviewers. Any product that may be evaluated in this article, or claim that may be made by its manufacturer, is not guaranteed or endorsed by the publisher.
